# Metastatic Non-Small-Cell Lung Cancer in the Setting of Unilateral Agenesis of the Left Pulmonary Artery: A Case Report and Comprehensive Literature Review

**DOI:** 10.1155/2019/4752835

**Published:** 2019-04-17

**Authors:** John Agzarian, Jakub Kadlec, Lori Whitehead, Yaron Shargall

**Affiliations:** ^1^McMaster University, Faculty of Health Sciences, Department of Surgery, St. Joseph's Healthcare Hamilton, T2105-50 Charlton Ave East, Hamilton, ON, Canada L8N 4A6; ^2^Norfolk and Norwich University Hospital Faculty of Medicine, Department of Surgery, Colney Lane, Norwich NR4 7UY, UK; ^3^McMaster University, Faculty of Health Sciences, Department of Medicine, Firestone Institute for Respiratory Health, St. Joseph's Healthcare Hamilton, T2105-50 Charlton Ave East, Hamilton, ON, Canada L8N 4A6

## Abstract

Unilateral absence of the pulmonary artery (UAPA) represents a rare condition that is often associated with cardiac congenital abnormalities but can also be relatively asymptomatic and indolent. There is a lack of consensus regarding the management of UAPA. However, in the setting of associated complications and ongoing infection, pulmonary resection is advocated. Although rare, the association between UAPA and bronchogenic carcinoma has been previously reported in seven published cases. In the majority of these, anatomic lung resection (most commonly with pneumonectomy) was curative. We present the first reported case of ipsilateral metastatic non-small-cell lung cancer- (NSCLC-) associated UAPA in a 47-year-old patient with ventilator-dependent hypoxic respiratory failure and bronchorrhea, who had been lost to follow-up for 8 years. Initial investigations did not yield evidence of malignancy, and confirmation of metastatic disease was made intraoperatively at the time of thoracotomy. The findings demonstrated evidence of diffuse metastatic pleural disease with lymphangitic carcinomatosis and superimposed infection. The patient was palliated and passed away shortly thereafter. In the setting of UAPA, clinicians should have a high index of suspicion for the possibility of malignancy, and if proven, they should consider early resection following appropriate staging.

## 1. Introduction

Unilateral absence of the pulmonary artery (UAPA) is a rare condition that is twice as common on the right side [1], with an overall prevalence of 1 in 200,000 to 1 in 300,000 [2, 3]. It was first described in 1868 [4], with 352 cases reported in the literature—237 of which were associated with cardiac anomalies [1, 5]. In the absence of associated cardiac abnormalities, isolated UAPA might remain relatively indolent with delayed diagnosis being made later in adulthood due to symptoms of exertional dyspnea (18-40%), recurrent respiratory infections (37%), pulmonary hypertension (19-44%), and hemoptysis (20%) [6–10]. UAPA is a congenital phenomenon resulting from the involution of the proximal sixth aortic arch with a persistent connection of the intrapulmonary pulmonary artery (PA) with the distal sixth aortic arch [11]. Collateral blood supply (aortopulmonary collateral vessels) to the affected ipsilateral lung usually arises from bronchial, intercostal, diaphragmatic, subclavian, and coronary arteries [3, 8, 12]. In the setting of isolated noncardiac-associated UAPA, treatment is indicated for symptomatic control of bleeding and/or infection [11]. This is often accomplished via pneumonectomy, closure of selected collateral arteries, embolization, or PA anastomosis [13–16]. Treatment is not necessary in asymptomatic cases, but follow-up should be undertaken at a regular basis [6].

The association between pulmonary artery agenesis and primary lung malignancy has been previously reported [17–22]. Often diagnosis of UAPA is made secondary to minor symptoms or incidentally [17]. The following is a unique case report of a presentation of UAPA with associated metastatic NSCLC that was nonresectable at the time of thoracotomy.

## 2. Case Presentation

**Figure 1:**
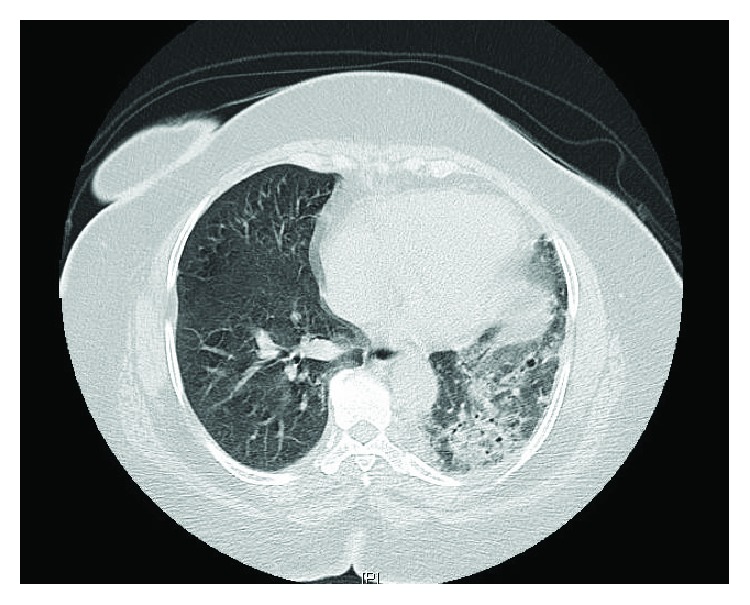
Axial CT of the chest with consolidative changes to the left lung and hypertrophied bronchial arteries.

**Figure 2:**
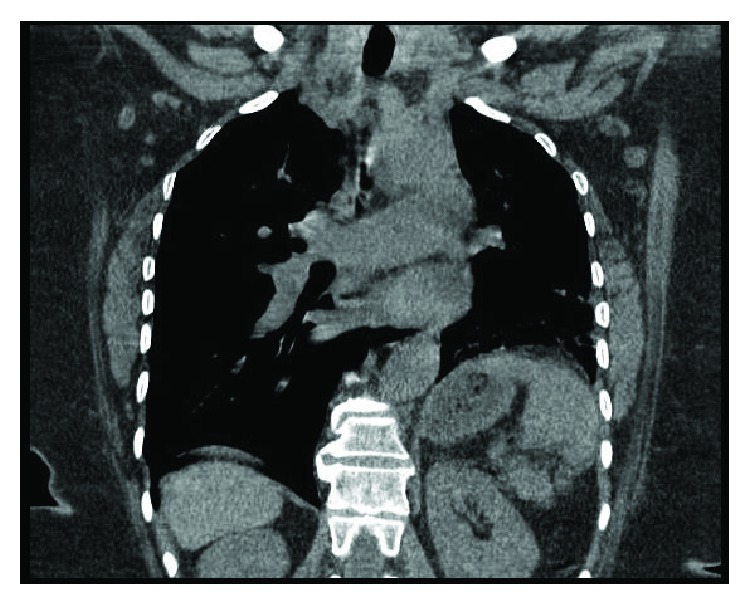
Coronal CT chest demonstrated marked enlargement of the right pulmonary artery with absence of the left pulmonary artery.

A 47-year-old HIV-negative female from northeast Africa was initially seen in consultation by the pulmonology service in 2007 for evaluation of positive TB skin test, abnormal chest X-ray (CXR) findings, and increasing symptoms of productive cough. She was a life-long nonsmoker, and her past medical history was significant for long-standing hypertension, osteoarthritis of the knee, and obesity. At the time, CXR demonstrated a density in the posterior left lower lobe and a poorly defined left hemidiaphragm. Bronchoscopy showed no obvious endobronchial abnormalities noted. Washings from the left upper and lower lobes consisted of negative cultures and negative acid-fast bacilli (AFB) analysis. Cytological analysis yielded a few multinucleated giant cells. CT of the chest demonstrated an enlarged right pulmonary artery and a slightly enlarged right atrium, with associated radiographic abnormalities of the left lung, including multifocal areas of consolidation, subpleural ground glass opacities and reticulation, and bronchiectasis. More notably, there was evidence of hypertrophied bronchial arteries providing collateral circulation to the left lung (Figures 1 and 2).

**Figure 3:**
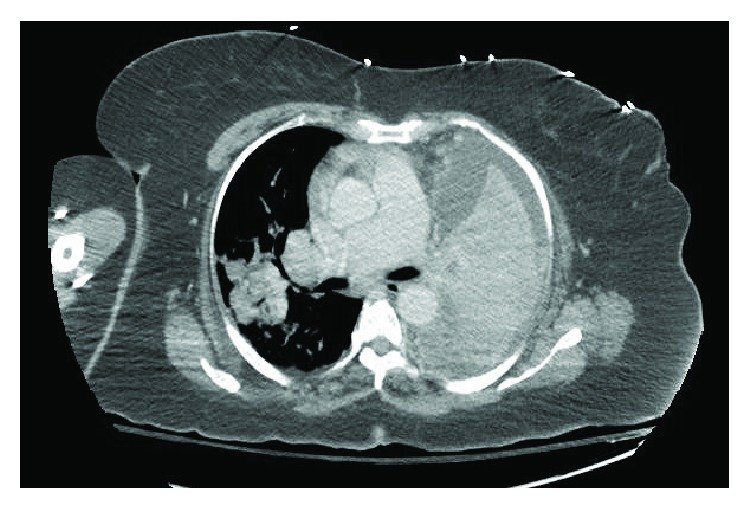
Extensive consolidation of the left lung with moderate to large pleural effusion and worsening consolidation of the right lung.

**Figure 4:**
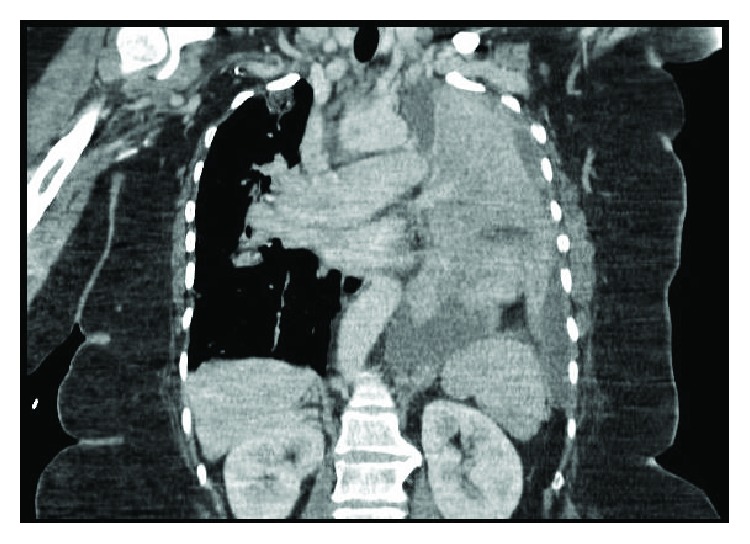
Coronal views with clear complete consolidation of the left lung, left pleural effusion, and worsening consolidative and nodular changes of the right lung.

The patient was lost to follow-up and seen again in 2015, at the age of 55, for evaluation of chronic pulmonary infiltrates, accompanied by symptoms of fever, cough, copious watery sputum production, low-volume hemoptysis, decreased exercise tolerance, and left-sided chest pain. Repeat CT scan examination confirmed absence of the left pulmonary artery with near complete consolidation of the left lung, as well as left-sided mediastinal shift and a small left pleural effusion. The right lung contained multifocal nodular opacities in all lobes (Figures 3 and 4). Given the radiographic and clinical changes, the patient was thought to have a primary infectious process (likely MTB complex) and was placed on respiratory isolation. Repeat bronchoscopy was performed with no growth of tuberculosis or other infectious agent and persistently negative AFB. The presumed diagnosis at that time was infectious vs. inflammatory in nature, with a remote possibility of malignancy still being considered. A thoracic surgical consultation was sought out, for consideration of open lung biopsy as a means of ruling out malignancy and to consider left pneumonectomy for symptom control.

The patient's symptoms ultimately continued to increase, requiring admission to a hospital for supplemental oxygen delivery. Transthoracic and surgical biopsies were deemed to be too highly risky and were ultimately not pursued. Despite aggressive medical therapy and broad spectrum antibiotic coverage, she required transfer to the intensive care unit due to hypoxic respiratory failure. Multidisciplinary conferencing concluded that the necrotic left lung was the primary culprit in the patient's decline, with acute chronic infection likely related to right lung contamination being the most likely diagnosis. The patient continued to decline clinically. She developed multisystem organ failure including respiratory compromise leading to intubation and mechanical ventilation, hypotension requiring ionotropic support, and worsening renal function. Accordingly, the decisive treatment option was for an emergent high-risk left pneumonectomy with consideration of preoperative embolization of collateral hypertrophied bronchial vessels. Preoperative CT investigation demonstrated a consolidated necrotic left lung, with moderate left pleural effusion and areas of thickening of the left pleura. On cardiopulmonary testing, echocardiogram revealed left-sided ejection fraction of 65-70%, with preserved right ventricular systolic function, without any significant tricuspid regurgitation. Quantitative lung ventilation-perfusion scanning identified 1% ventilation and perfusion to the left lung, with >90% right-sided lung perfusion. The patient and her family were quoted a near 50-90% surgical mortality, in the face of imminent death without intervention.

The patient was taken to the operating room and placed in the right lateral decubitus position, and an endobronchial blocker was placed through her single-lumen endotracheal tube and positioned in the left mainstem bronchus. A posterolateral thoracotomy was performed, after harvesting a serratus anterior muscle flap. Upon entry into the pleural space, it was evident that the left lung was cemented to the parietal pleura with dense thick adhesions. Minimal dissection was undertaken in order to identify an area of the pleural cavity that was free. Five hundred milliliters of serous pleural fluid was drained. Additionally, there was evidence of multiple small pleural nodules dispersed throughout the chest cavity and the hilum. Frozen section analysis of these pleural deposits confirmed the presence of metastatic adenocarcinoma, confirming the presence of stage IV lung cancer. As such, the planned left pneumonectomy was aborted and the chest was closed with an indwelling chest tube.

She was transferred back to the ICU with continued mechanical ventilation. The working diagnosis at that point was diffuse metastatic lung cancer with lymphangitic carcinomatosis and superimposed infection. After the family was informed of the operative findings, a palliative approach was undertaken. Comfort measures were ensured. Mechanical ventilation was weaned off as care was being withdrawn, and the patient peacefully passed away.

Pleural fluid analysis identified the presence of malignant cells suggestive of adenocarcinoma. Final pathologic analysis of intraoperative specimens demonstrated pleural tissue comprised of tall columnar tumor cells with focal necrosis, infiltrated by adenocarcinoma (thyroid transcription factor-1 positive) with predominantly papillary features. Epidermal growth factor receptor (EGFR) mutation was not identified (wild-type allele) by polymerase chain reaction (PCR) analysis using the EntroGen EGFR Kit Exons 18, 19, 20, and 21. In addition, ALK rearrangement was not identified.

## 3. Literature Review and Discussion

Several interesting factors contribute to the diagnostic complexity of this case. Attempts at invasive testing were made in order to determine the possibility of malignancy. Due to patient delays and worsening clinical status, this ultimately never occurred. Similarly, as a consequence of the patient's deteriorating critical state, appropriate lung cancer staging investigation was not performed, including positron emission topography (PET) scan, invasive mediastinal staging, and cranial imaging. The diagnostic yield of these investigations in the clinical context is uncertain. Overall, the preoperative determination of malignancy was challenged by two clinical features. Firstly, more than three separate bronchoscopic evaluations with lavages and brushings did not demonstrate the presence of malignancy—a rare phenomenon in a patient with bronchorrhea and such diffuse disease. Secondly, the protracted course of the presentation (spanning over 8 years) would be unusual for a malignant process.

This case represents the only report in the literature of diffuse metastatic lung cancer associated with UAPA and highlights the challenge of determining a malignant diagnosis in the setting of consolidated lung secondary to pulmonary arterial agenesis. To our knowledge, this is also the only case of UAPA associated lung cancer in a lifelong nonsmoker. To date, there have been 6 reported cases of UAPA with secondarily associated non-small-cell lung cancer (NSCLC) [17–22]. In all but one case, the primary lung malignancy was completely resected via pneumonectomy or lobectomy. The association was first reported in 1975 by Mancebo and Wanner, based on the presence of metastatic undifferentiated carcinoma in mediastinal lymph nodes as noted on mediastinoscopy. Accordingly, pulmonary resection was not undertaken [19]. Roman et al. in 1995 and Makdisi et al. in 2015 both reported successful pneumonectomy for primary NSCLC with ipsilateral UAPA [18, 20]. The latter was the first report of multifocal lung cancer in the setting of pulmonary artery agenesis. In 2011, Anstadt et al. presented the only case of ipsilateral lobectomy for lung cancer in the context of UAPA [22]. In contrast, Ito et al. reported the only case of postradiotherapy contralateral pulmonary resection (right middle lobectomy) of the normal lung in the setting of left-sided UAPA, outlining the possibility of surgical intervention on the nonaffected lung [17].

The causal association between UAPA and lung cancer has yet to be completely defined. Several plausible explanations have been provided. The possibility of structural abnormalities in a hypoplastic underperfused lung serving as a nidus for malignant transformation has been previously suggested [21]. More recently, the role of chronic hypoxia as an impetus for DNA damage and the development of lung malignancy has been highlighted [23]. A hypoxic state at the core of a hypoperfused lung can lead to the release of reactive oxygen species (ROS), which in turn induce cell proliferation via the activation of the EGFR pathway [24, 25].

## 4. Conclusion

Taken together, this case depicts another rare example of isolated UAPA with associated primary lung malignancy. It represents the seventh reported case in the literature and is the only presentation associating pulmonary arterial agenesis with metastatic lung cancer leading to acute respiratory failure and death. When faced with the uncommon scenario of UAPA, clinicians must actively consider the possibility of concomitant malignancy with expedited resection (when appropriate) after complete diagnostic confirmation and staging.

## References

[1] Bockeria L. A., Makhachev O. A., Khiriev T. K., Abramyan M. A. (2011). Congenital isolated unilateral absence of pulmonary artery and variants of collateral blood supply of the ipsilateral lung. *Interactive CardioVascular and Thoracic Surgery*.

[2] Bouros D., Pare P., Panagou P., Tsintiris K., Siafakas N. (1995). The varied manifestation of pulmonary artery agenesis in adulthood. *Chest*.

[3] Kadir I. S., Thekudan J., Dheodar A., Jones M. T., Carroll K. B. (2002). Congenital unilateral pulmonary artery agenesis and aspergilloma. *The Annals of Thoracic Surgery*.

[4] Fraentzel O. (1868). Ein fall von abnormer communication der aorta mit der arteria pulmonalis. *Archiv für Pathologische Anatomie und Physiologie und für Klinische Medicin*.

[5] Finney J. O., Finchum R. N. (1972). Congenital unilateral absence of the left pulmonary artery with right aortic arch arid a normal conus. *Southern Medical Journal*.

[6] Shakibi J. G., Rastan H., Nazarian I., Paydar M., Aryanpour I., Siassi B. (1978). Isolated unilateral absence of the pulmonary artery: review of the world literature and guidelines for surgical repair. *Japanese Heart Journal*.

[7] Bahler R. C., Carson P., Traks E., Levene A., Gillespie D. (1969). Absent right pulmonary artery: Problems in diagnosis and management. *The American Journal of Medicine*.

[8] Ten Harkel A. D. J., Blom N. A., Ottenkamp J. (2002). Isolated unilateral absence of a pulmonary artery. *Chest*.

[9] Griffin N., Mansfield L., Redmond K. C. (2007). Imaging features of isolated unilateral pulmonary artery agenesis presenting in adulthood: a review of four cases. *Clinical Radiology*.

[10] Krall W. R., Ploy-Song-Sang Y. (1980). Unilateral pulmonary artery aplasia presenting with chest pain and pleural effusion. *Southern Medical Journal*.

[11] Kruzliak P., Syamasundar R. P., Novak M., Pechanova O., Kovacova G. (2013). Unilateral absence of pulmonary artery: pathophysiology, symptoms, diagnosis and current treatment. *Archives of Cardiovascular Diseases*.

[12] Gupta K., Livesay J. J., Lufschanowski R. (2001). Absent right pulmonary artery with coronary collaterals supplying the affected lung. *Circulation*.

[13] Moreno-Cabral R. J., McNamara J., Reddy V. J., Caldwell P. (1991). Unilateral absent pulmonary artery: surgical repair with a new technique. *The Journal of Thoracic and Cardiovascular Surgery*.

[14] Canver C. C., Pigott J. D., Mentzer R. M. (1991). Neonatal pneumonectomy for isolated unilateral pulmonary artery agenesis. *The Annals of Thoracic Surgery*.

[15] Sreeram N., Asante-Korang A., Ladusans E. (1992). Distal ductal origin of the right pulmonary artery: prospective diagnosis and primary repair in infancy. *International Journal of Cardiology*.

[16] Ohta T. (1983). Congenital absence of the right pulmonary artery — a case report and review. *The Tokai Journal of Experimental and Clinical Medicine*.

[17] Ito M., Yamashita Y., Harada H., Omori K. I. (2010). Unilateral absence of the left pulmonary artery accompanied by right lung cancer. *The Annals of Thoracic Surgery*.

[18] Roman J., Jones S. (1995). Case report: congenital absence of the left pulmonary artery accompanied by ipsilateral emphysema and adenocarcinoma. *The American Journal of the Medical Sciences*.

[19] Mancebo A., Wanner A. (1975). Lung tumor in a patient with congenital unilateral hypoplasia of the pulmonary artery. *Chest*.

[20] Makdisi G., Edell E. S., Maleszewski J. J., Molina J. R., Deschamps C. (2015). Pulmonary artery agenesis associated with emphysema and multiple invasive non-small cell lung cancers. *The Annals of Thoracic Surgery*.

[21] Watanabe Y., Shibuya J., Handa M. (2015). Unilateral absence of the right pulmonary artery accompanied by right lung cancer. *The Annals of Thoracic Surgery*.

[22] Anstadt M. P., Wozniak C. J., Kathula S. S. K., Sarodia B. D. (2011). Lobectomy for lung carcinoma with ipsilateral pulmonary artery agenesis. http://www.atsjournals.org/doi/abs/10.1164/ajrccm-conference.2011.183.1_MeetingAbstracts.A3836.

[23] Srujana K., Begum S. S., Rao K. N., Devi G. S., Jyothy A., Prasad M. H. (2010). Application of the comet assay for assessment of oxidative DNA damage in circulating lymphocytes of tetralogy of Fallot patients. *Mutation Research/Fundamental and Molecular Mechanisms of Mutagenesis*.

[24] Lemjabbar-Alaoui H., Sidhu S. S., Mengistab A., Gallup M., Basbaum C. (2011). TACE/ADAM-17 phosphorylation by PKC-epsilon mediates premalignant changes in tobacco smoke-exposed lung cells. *PLoS One*.

[25] Franovic A., Gunaratnam L., Smith K., Robert I., Patten D., Lee S. (2007). Translational up-regulation of the EGFR by tumor hypoxia provides a nonmutational explanation for its overexpression in human cancer. *Proceedings of the National Academy of Sciences of the United States of America*.

